# Supplementary material for: Machine Learning Accelerates Crystallization for Structure Determination
Source: Angew Chem Int Ed Engl. 2026 May 4;65(25):e1218503. doi: 10.1002/anie.1218503 (PMC13266958; doi:10.1002/anie.1218503)

checkCIF/PLATON report

Structure factors have been supplied for datablock(s) lcz008

THIS REPORT IS FOR GUIDANCE ONLY. IF USED AS PART OF A REVIEW PROCEDURE FOR PUBLICATION, IT SHOULD NOT REPLACE THE EXPERTISE OF AN EXPERIENCED CRYSTALLOGRAPHIC REFEREE.

No syntax errors found. CIF dictionary Interpreting this report

Datablock: lcz008

```
Bond precision:   C-C = 0.0199 A          Wavelength=1.54184

Cell:            a=10.1664(3)      b=12.0268(4)      c=13.4008(4)
                 alpha=72.917(3)   beta=75.561(3)    gamma=78.910(2)
Temperature:     100 K

                  Calculated                   Reported
Volume            1504.14(9)                   1504.14(9)
Space group       P -1                         P -1
Hall group        -P 1                         -P 1
Moiety formula    C22 H13 Ag3 F18 N6 O         C15 H3 Ag3 F18 N6, C7 H10 O
Sum formula       C22 H13 Ag3 F18 N6 O         C22 H13 Ag3 F18 N6 O
Mr                1042.99                      1042.99
Dx,g cm-3         2.303                        2.303
Z                 2                            2
Mu (mm-1)         16.872                       16.872
F000              996.0                        996.0
F000'             1000.94
h,k,lmax          12,15,17                     12,15,16
Nref              6432                         6095
Tmin,Tmax         0.052,0.067                  0.414,1.000
Tmin'             0.008

Correction method= # Reported T Limits: Tmin=0.414 Tmax=1.000
AbsCorr = MULTI-SCAN
```

Data completeness= 0.948   Theta(max)= 78.130

R(reflections)= 0.0788(4794)   wR2(reflections)= 0.1883(6095)

S = 1.230   Npar= 451

The following ALERTS were generated. Each ALERT has the format
test-name_ALERT_alert-type_alert-level.
Click on the hyperlinks for more details of the test.

Alert level C

```
PLAT042_ALERT_1_C Calc. and Reported MoietyFormula Strings  Differ     Please Check
              Calc: C22 H13 Ag3 F18 N6 O
              Rep.: C15 H3 Ag3 F18 N6, C7 H10 O
PLAT213_ALERT_2_C Atom F00S            has ADP max/min Ratio .....        3.1 prolat
PLAT213_ALERT_2_C Atom F014            has ADP max/min Ratio .....        3.1 prolat
PLAT234_ALERT_4_C Large Hirshfeld Difference F004     --C013     .       0.18 Ang.
PLAT234_ALERT_4_C Large Hirshfeld Difference F00L     --C011     .       0.17 Ang.
PLAT234_ALERT_4_C Large Hirshfeld Difference F00N     --C011     .       0.21 Ang.
PLAT234_ALERT_4_C Large Hirshfeld Difference C010     --C01D     .       0.20 Ang.
PLAT241_ALERT_2_C High   'MainMol' Ueq as Compared to Neighbors of       C019 Check
PLAT241_ALERT_2_C High   'MainMol' Ueq as Compared to Neighbors of       C01B Check
PLAT242_ALERT_2_C Low    'MainMol' Ueq as Compared to Neighbors of       C010 Check
PLAT342_ALERT_3_C Low Bond Precision on  C-C Bonds ..............     0.0199 Ang.
PLAT906_ALERT_3_C Large K Value in the Analysis of Variance ......     16.326 Check
PLAT906_ALERT_3_C Large K Value in the Analysis of Variance ......      3.295 Check
PLAT911_ALERT_3_C Missing FCF Refl Between Thmin & STh/L=    0.600         49 Report
                0  1  0,   2  2  0, -11  3  0, -10  5  0,  -1 -5  1,  10 -4  1,
                5 -3  1,  11 -3  1,  11  0  1,   1  1  1,  -8  7  1,  10 -3  2,
              -11 -2  2,   7  4  2,  11  6  2,  -9 -8  3,  10 -3  3, -10 -2  4,
               12  1  4,  10  2  4,  11  3  4,  11  4  4,   4  6  4,  -8  8  4,
                0 -4  5,  -9 -3  5,  11  0  5,  11  1  5,  12  1  5,  12  2  5,
                   (   19 More Missing: see the .ckf listing file)
PLAT934_ALERT_3_C Number of (Iobs-Icalc)/Sigma(W) > 10 Outliers ..          1 Check
               10  2  5,
PLAT971_ALERT_2_C Check Calcd Resid. Dens.  1.05Ang From F00V           1.53 eA-3
```

Alert level G

```
PLAT002_ALERT_2_G Number of Distance or Angle Restraints on AtSite         22 Note
PLAT003_ALERT_2_G Number of Uiso or U(i,j)  Restrained non-H-Atoms          3 Report
PLAT083_ALERT_2_G SHELXL Second Parameter in WGHT  Unusually Large      26.86 Why ?
PLAT172_ALERT_4_G The CIF-Embedded .res File Contains DFIX Records          3 Report
PLAT177_ALERT_4_G The CIF-Embedded .res File Contains DELU Records          2 Report
PLAT192_ALERT_3_G A Non-default DELU Restraint Value for SecondPar     0.0200 Report
PLAT192_ALERT_3_G A Non-default DELU Restraint Value for SecondPar     0.0200 Report
PLAT242_ALERT_2_G Low    'MainMol' Ueq as Compared to Neighbors of       C00Y Check
PLAT242_ALERT_2_G Low    'MainMol' Ueq as Compared to Neighbors of       C011 Check
PLAT242_ALERT_2_G Low    'MainMol' Ueq as Compared to Neighbors of       C012 Check
PLAT242_ALERT_2_G Low    'MainMol' Ueq as Compared to Neighbors of       C013 Check
PLAT242_ALERT_2_G Low    'MainMol' Ueq as Compared to Neighbors of       C016 Check
PLAT242_ALERT_2_G Low    'MainMol' Ueq as Compared to Neighbors of       C018 Check
PLAT434_ALERT_2_G Short Inter HL..HL Contact F00I     ..F00O     .       2.79 Ang.
```

```
                                                 1-x,1-y,1-z  =      2_666 Check
PLAT720_ALERT_4_G Number of Unusual/Non-Standard Labels ..........         63 Note
              Ag01    Ag02    Ag03    F004    O005    F006    F007    N008
              F009    F00A    F00B    N00C    N00D    F00E    N00F    F00G
              N00H    F00I    N00J    F00K    F00L    F00M    F00N    F00O
              F00P    C00Q    C00R    H00R    F00S    C00T    C00U    H00U
              F00V    C00W    C00X    C00Y    C00Z    C010    C011    C012
              C013    F014    C015    C016    C017    H017    C018    C019
              H01A    H01B    C01A    H01C    C01B    H01D    H01E    C01C
              H01F    H01G    C01D    H01H    C01E    H01I    H01J
PLAT793_ALERT_4_G Model has Chirality at C01A        (Centro SpGr)          S Verify
PLAT793_ALERT_4_G Model has Chirality at C01D        (Centro SpGr)          R Verify
PLAT860_ALERT_3_G Number of Least-Squares Restraints ............          23 Note
PLAT883_ALERT_1_G Absent Datum for _atom_sites_solution_primary ..     Please Do !
PLAT912_ALERT_4_G Missing # of FCF Reflections Above STh/L=  0.600        287 Note
PLAT913_ALERT_3_G Missing # of Very Strong Reflections in FCF ....          1 Note
               2  2  0,
PLAT933_ALERT_2_G Number of HKL-OMIT Records in Embedded .res File          6 Note
              -11 -5  3, -11 -2  2, -10 -2  4,  10  2  4,  11  4  4,  11  4  7,
PLAT941_ALERT_3_G Average HKL Measurement Multiplicity ..........         2.2 Low
PLAT969_ALERT_5_G The 'Henn et al.' R-Factor-gap value ..........       3.603 Note
              Predicted wR2: Based on SigI**2  5.23 or SHELX Weight 15.31
PLAT978_ALERT_2_G Number C-C Bonds with Positive Residual Density.          0 Info
```

```
   0 ALERT level A = Most likely a serious problem - resolve or explain
   0 ALERT level B = A potentially serious problem, consider carefully
  16 ALERT level C = Check. Ensure it is not caused by an omission or oversight
  25 ALERT level G = General information/check it is not something unexpected

   2 ALERT type 1 CIF construction/syntax error, inconsistent or missing data
  18 ALERT type 2 Indicator that the structure model may be wrong or deficient
  10 ALERT type 3 Indicator that the structure quality may be low
  10 ALERT type 4 Improvement, methodology, query or suggestion
   1 ALERT type 5 Informative message, check
```

Validation response form

Please find below a validation response form (VRF) that can be filled in and pasted into your CIF.

```
# start Validation Reply Form
_vrf_PLAT042_lcz008
;
PROBLEM: Calc. and Reported MoietyFormula Strings  Differ     Please Check
RESPONSE: ...
;
_vrf_PLAT213_lcz008
;
PROBLEM: Atom F00S            has ADP max/min Ratio .....        3.1 prolat
RESPONSE: ...
;
_vrf_PLAT234_lcz008
;
PROBLEM: Large Hirshfeld Difference F004     --C013     .       0.18 Ang.
RESPONSE: ...
;
```

```
_vrf_PLAT241_lcz008
;
PROBLEM: High   'MainMol' Ueq as Compared to Neighbors of       C019 Check
RESPONSE: ...
;
_vrf_PLAT242_lcz008
;
PROBLEM: Low    'MainMol' Ueq as Compared to Neighbors of       C010 Check
RESPONSE: ...
;
_vrf_PLAT342_lcz008
;
PROBLEM: Low Bond Precision on  C-C Bonds ...............     0.0199 Ang.
RESPONSE: ...
;
_vrf_PLAT906_lcz008
;
PROBLEM: Large K Value in the Analysis of Variance ......     16.326 Check
RESPONSE: ...
;
_vrf_PLAT911_lcz008
;
PROBLEM: Missing FCF Refl Between Thmin & STh/L=    0.600         49 Report
RESPONSE: ...
;
_vrf_PLAT934_lcz008
;
PROBLEM: Number of (Iobs-Icalc)/Sigma(W) > 10 Outliers ..          1 Check
RESPONSE: ...
;
_vrf_PLAT971_lcz008
;
PROBLEM: Check Calcd Resid. Dens.  1.05Ang From F00V           1.53 eA-3
RESPONSE: ...
;
# end Validation Reply Form
```

It is advisable to attempt to resolve as many as possible of the alerts in all categories. Often the minor alerts point to easily fixed oversights, errors and omissions in your CIF or refinement strategy, so attention to these fine details can be worthwhile. It is up to the individual to critically assess their own results and, if necessary, seek expert advice.

PLATON version of 17/09/2025; check.def file version of 17/09/2025

---

duplicate check

No duplication found

Datablock lcz008 - ellipsoid plot

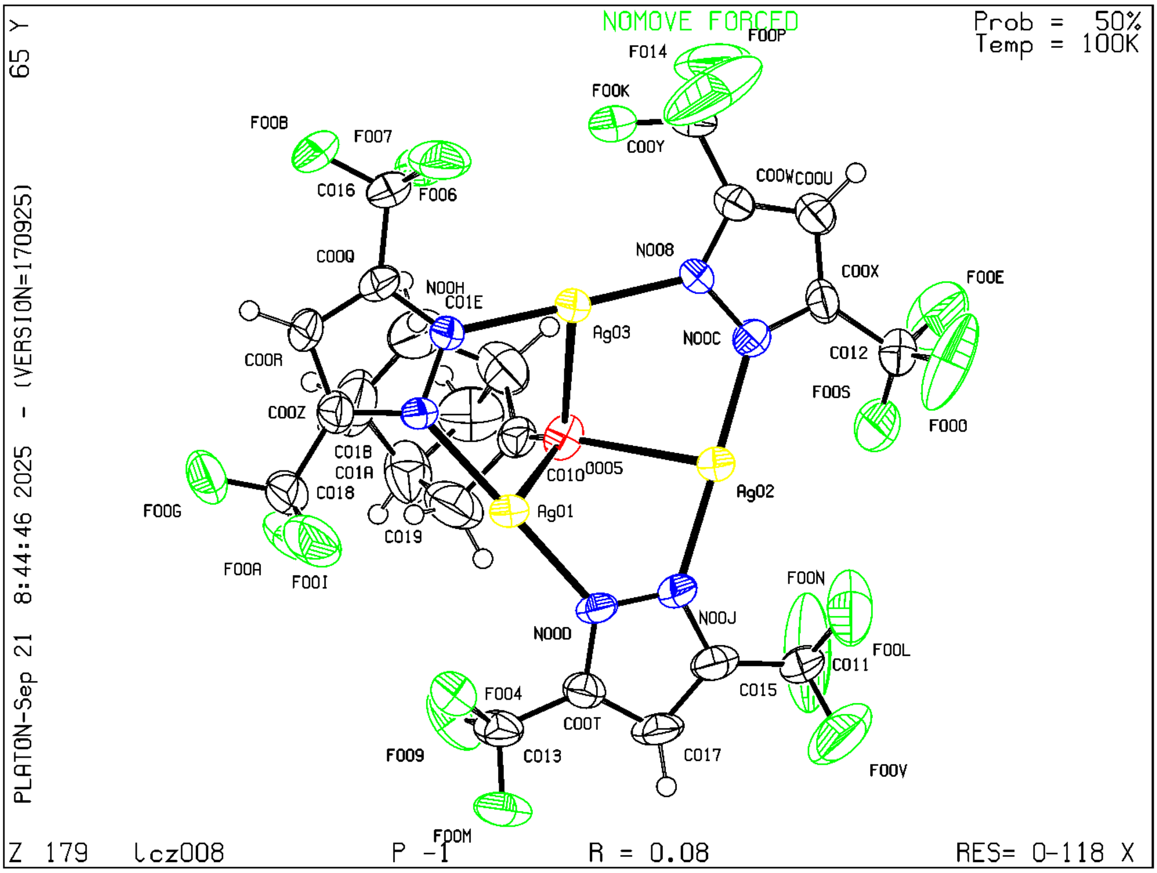

Supplement: Supplementary file 2 — Supporting File 2: anie72462‐sup‐0002‐DataFile.zip. [file ANIE-65-e1218503-s002.zip › supplementary_materials_crystallographic_data submit.zip!/cif_and_checkcif(1-114)/Ag3Pz3_8/Ag3Pz3_8_checkcif.pdf]
